# Referral Patterns for Patients with Nonalcoholic Fatty Liver Disease

**DOI:** 10.3390/jcm10030404

**Published:** 2021-01-21

**Authors:** Elias Badal Rashu, Mikkel Parsberg Werge, Liv Eline Hetland, Anders Ellekaer Junker, Majken Karoline Jensen, Lise Lotte Gluud

**Affiliations:** 1 Gastro Unit, Copenhagen University Hospital Hvidovre, 2650 Hvidovre, Denmark; elias.badal.rashu@regionh.dk (E.B.R.); mikkel.parsberg.werge@regionh.dk (M.P.W.); liv.eline.bjoerge.hetland@regionh.dk (L.E.H.); anders.ellekaer.junker.01@regionh.dk (A.E.J.); 2 Section of Epidemiology, Department of Public Health, University of Copenhagen, 1356 Copenhagen, Denmark; maje@sund.ku.dk

**Keywords:** cohort study, non-alcoholic steatohepatitis, cirrhosis, gastroenterology, hepatology, type 2 diabetes, metabolic syndrome

## Abstract

The incidence of nonalcoholic fatty liver disease (NAFLD) is rapidly increasing. This study evaluates the referral pattern of patients with NAFLD. A cohort study evaluating all patients with NAFLD referred to a single Gastroenterology Department from January 2017 to June 2020. Electronic patient referral letters were reviewed, and patients with NAFLD were diagnosed using standardized tests as part of a prospective cohort study. Predictors of nonalcoholic steatohepatitis (NASH) with significant (≥F2) fibrosis were evaluated in logistic regression analyses. In total, 323 (18.6%) of 1735 patients referred to the Gastro Unit during the study period were diagnosed with NAFLD. Patients were referred from general practitioners (62.5%) or other hospital departments (37.5%). Most referral letters included information suggesting a possible diagnosis of NAFLD (patient history, blood tests, or diagnostic imaging) or used the nonspecific general diagnosis suspected disease (Z.038). Out of 110 patients referred for a liver biopsy, 71 (22%) had NASH with significant fibrosis (F2 *n* = 39, F3 *n* = 19, F4 *n* = 13). Thirty-nine of these patients were referred from the primary sector. A logistic regression analysis (adjusted for age and gender) including all 323 patients showed that type 2 diabetes was the only significant independent predictor of NASH with fibrosis.

## 1. Introduction

Nonalcoholic fatty liver disease (NAFLD) is the most common liver disease in Western Countries, affecting 15 to 30% of the general population [1,2,3,4,5,6]. NAFLD covers a wide spectrum from simple steatosis (nonalcoholic fatty liver) to nonalcoholic steatohepatitis (NASH). NASH may lead to fibrosis and eventually cirrhosis as well as an increased risk of hepatocellular carcinoma [7]. Simple steatosis may also lead to fibrosis, although this is rare. Both simple steatosis and NASH are associated with an increased risk of cardiovascular disease [8,9]. Although NAFLD may occur in patients with a normal glucose tolerance, the risk of NASH is closely linked with obesity and type 2 diabetes. Accordingly, the prevalence of NASH is increasing rapidly [5]. NASH-related cirrhosis is predicted to become the most common indication for liver transplantation in several countries and poses a considerable clinical and economic burden in Europe and the United States [10].

Lifestyle interventions are the gold standard for all patients with NAFLD or NASH [1,2,11]. Medical interventions are not recommended for patients with simple steatosis but should be considered in patients with NASH and significant fibrosis. The identification of patients with NASH and significant fibrosis is therefore essential. Noninvasive markers such as transient elastography or fibrosis markers may be used to evaluate the baseline risk of NASH, but the diagnosis still requires a histological assessment. Liver biopsy remains the only diagnostic procedure that reliably differentiates simple steatosis from NASH [1,12]. Performing a liver biopsy is expensive and associated with a risk of adverse events. A histological assessment is recommended if fibrosis is suspected and to exclude a differential diagnosis. Due to the lack of accurate biomarkers, the identification and referral of patients with NASH and significant fibrosis is difficult. We therefore undertook a study evaluating referral patterns in patients with NAFLD.

## 2. Materials and Methods

This study examined all referrals to the Gastro Unit, Copenhagen University Hospital Hvidovre, Denmark from January 2017 to June 2020. The Gastro Unit is the largest department specializing in Gastroenterology and Hepatology in the capital region of Denmark with a catchment population of 515.000 persons. This paper describes a substudy of the Fatty Liver Disease in Nordic Countries (FLINC) study (Clinicaltrials.gov NCT04340817, H-17029039). The FLINC study is a prospective cohort study evaluating biomarkers in patients with NAFLD. All patients referred with suspected NAFLD underwent a standardized diagnostic program. Initial blood tests included standard biochemistry, liver blood tests, and metabolic markers. Differential diagnoses were carefully evaluated, including possible alcoholic liver disease, drug-induced liver injury, chronic viral hepatitis, autoimmune liver diseases, haemochromatosis, and other less common causes. The diagnostic imaging consisted of an abdominal ultrasound and a fibroscan with the registration of the liver stiffness measurement (median) and continuous attenuation parameter (CAP) values. Patients suspected of NASH with significant fibrosis and who consented underwent a percutaneous or transjugular liver biopsy. Biopsies were assessed according to international recommendations in a standardized manner. NASH were diagnosed based on the sum of scores for steatosis, hepatocellular ballooning, and lobular inflammation as well as the degree of fibrosis from F0 (simple steatosis) to F4 (cirrhosis) [12,13].

We reviewed the electronic patient records and referral letters for all patients referred to the Gastro Unit with possible NAFLD. The system is used by all hospital Departments in the Capital Region of Denmark. Information about previous blood tests were available for all patients. Data were gathered in an electronic database including the referral diagnosis according to the International Classification of Diseases, Tenth Revision (ICD10), patient characteristics, the rationale for the diagnosis, and the final diagnosis.

## 3. Results

### 3.1. Patient Characteristics

In total, 1735 patients with gastroenterological diseases were referred to the Gastro Unit during the study period, and 323 patients (18.6%) were diagnosed with NAFLD. None of the patients referred with a suspected diagnosis of NAFLD were diagnosed with other liver diseases. Patients with NAFLD were referred from general practitioners (*n* = 202, 62.5%) or other hospital departments (*n* = 101, 37.5%).

Forty-six patients (14.2%) were referred from other gastroenterology departments (medical *n* = 28, 23.1% or surgical *n* = 18, 14.9%). The remaining patients were referred from departments specializing in endocrinology (*n* = 24, 19.8%), cardiology (*n* = 8, 6.6%), rheumatology (*n* = 4, 1.2%), nephrology (*n* = 4, 1.2%), haematology (*n* = 4, 1.2%), or other specialties (pulmonary medicine, neurology, psychiatry, urology, dermatology, and emergency medicine (*n* = 31, 9.6%).

Among the 323 patients who were diagnosed with NAFLD, 89 (27.6%) were referred with the general diagnosis *suspected disease* (Z.038). The majority of these referrals were sent by general practitioners (*n* = 83, 93.3%). In 30% (*n* = 97) of the remaining referral letters, the ICD10 codes were *fatty (change of) liver* (K.76 including nonalcoholic fatty liver disease), *abnormal serum enzyme levels* (R.74), and *unspecified liver disease* (K.76). The remaining ICD10 diagnoses in the referral letters were cirrhosis (K.74), abdominal pain (R.10), hepatomegaly (R.16), and disorders of iron metabolism (E.83).

Most referral letters included information that patients had one or more signs or symptoms suggesting a possible diagnosis of NAFLD (Table 1).

In 220 of the letters (68.1%), information was included about elevated liver blood tests; 154 (47.7%) of these were sent from general practitioners. The latter supplied values of the alanine aminotransferase and/or gamma-glutamyl transferase levels. None measured aspartate aminotransferase or calculated the Fib-4 index. The second most common referral cause was abdominal ultrasounds showing evidence of hepatic steatosis (*n* = 187, 57.9%); most of these were also sent by general practitioners (*n* = 111, 34%). Other referrals included abdominal pain and discomfort (*n* = 72, 22.3%), prolonged fatigue (*n* = 32, 9.9%), and elevated ferritin levels (*n* = 27, 8.3%). Three patients (1%) were referred with decompensated cirrhosis diagnosed in the emergency department.

### 3.2. Information Available at Referral

At the time of referral, 202 patients (62.5%) had undergone abdominal ultrasonography; 129 of these patients were referred from general practitioners (39.9%) and had undergone ultrasonography at specialized radiologist clinics. The findings were described as mild to severe steatosis and hepatomegaly. Fourteen patients referred from other departments had undergone transient elastography (4.3%) with a mean LSM value of 9.3 kPa (±3.3) and CAP of 349.5 dB/m (±55). Thirty-four patients had possible steatosis or cirrhosis described on a Computed Tomography (CT) scan (*n* = 34, 10.5%) or magnetic resonance imaging (*n* = 12, 3.7%). Seventeen patients had undergone a liver biopsy and were referred with NASH and significant (at least F2) fibrosis (5.3%) from other gastroenterology departments.

Three patients were referred with a possible diagnosis of cirrhosis based on the macroscopic findings at nonliver-related surgery (gastrosurgical *n* = 2, gynaecology *n* = 1).

Several differential diagnoses were excluded before the referral of most patients (Table 2). A detailed patient history regarding alcohol intake was provided for 183 patients (56.7%); 108 of these were referred from general practitioners (33.4%). Drug-induced liver injury (DILI) was judged to be excluded in 70 referrals (21.7%) by written information about prescription and nonprescription medication. Almost half of the patients were tested for chronic viral hepatitis B and C before referral (*n* = 150, 46.4%), and 120 patients (37.2%) tested negative for haemochromatosis (normal transferrin saturation and serum ferritin levels). Autoimmune liver diseases (autoimmune hepatitis and primary biliary cholangitis) were excluded in 86 referrals (26.6%). A small proportion of patients were also tested for Wilson’s disease, coeliac disease, and alpha-1 antitrypsin deficiency before referral.

### 3.3. Diagnostic Assessment

Of the 323 patients with NAFLD, 50.5% were female, and the mean age at the time of referral was 47.5 years. Obesity (body mass index >30 kg/m^2^) was registered for 216 patients (66.9%) and hypertension for 119 patients (36.8%). Diagnostic blood tests revealed that 59.8% had dyslipidaemia and that 94 (40.5%) had type 2 diabetes. Most patients were asymptomatic. In total, 42 (13%) complained of abdominal pain, 89 (27.6%) of fatigue, and 17 (5.3%) of nausea. The initial assessments suggested that 132 (40.9%) had NASH with significant fibrosis. Twenty-two patients did not undergo a liver biopsy (6.8%). Ten of these patients (with repeated fibroscan values above 9 kPa) declined a liver biopsy, and 12 patients did not undergo a liver biopsy due to old age, comorbidities, or decompensated cirrhosis. In addition, a liver biopsy was available for 15 patients (4.6%) referred from other hospital departments (F2 *n* = 4, F3 *n* = 7, F4 *n* = 4). In total, 110 patients (34.1%) underwent a liver biopsy (percutaneous *n* = 65, or transjugular *n* = 35). Seventy-one patients (22%) had liver biopsies with significant fibrosis (F2 *n* = 39, F3 *n* = 19, F4 *n* = 13). The proportion of patients with significant fibrosis referred from the primary sector was 12.1% (*n* = 39), whereas 10% (*n* = 32) of those who underwent a liver biopsy showing NASH with significant fibrosis were referred from other hospital departments.

In the univariable logistic regression analysis adjusted for age and gender, only type 2 diabetes was significantly associated with NASH and significant fibrosis, with an odds ratio of 2.65 (95% CI 1.53 to 4.78) after adjusting for age and sex (Table 3). None of the remaining factors were statistically significant. None of the variables were significant in the multivariable-adjusted analysis (with all predictors entered).

## 4. Discussion

This study found that a relatively high proportion of patients referred with NAFLD had NASH with significant fibrosis. This is noteworthy since none were specifically assessed for Fib-4 scores or other validated scores evaluating the risk of fibrosis. A small proportion of patients were referred after an assessment with liver biopsies or transient elastography. Most patients were referred with elevated liver enzymes or diagnostic imaging suggesting steatosis.

Patients were referred from general practitioners or other hospital departments. Although a large proportion of patients with NAFLD are referred with a nonspecific ICD10 diagnosis, referral letters often contain information suggesting NAFLD. Several patients had nonspecific symptoms, and it is possible that these symptoms could influence the choice to refer patients for further assessment. However, our analyses confirmed that the symptoms were not associated with the severity of the disease.

Valid biomarkers are needed to identify patients with severe NASH in order to reduce the need for a histological assessment but also in order to improve referrals. The relative number of patients without significant fibrosis was high and is likely to be increasing. A previous study cohort study with analyses before and after the introduction of a pathway for the management of patients with NAFLD found that the use of a two-step algorithm combining the fibrosis-4 score and the enhanced liver fibrosis score significantly reduced the number of unnecessary referrals [14]. It is possible that the use of valid biomarkers can also reduce the risk of an under-referral of patients who are eligible for assessment and possible medical treatment at specialized departments.

In agreement with previous findings, type 2 diabetes was linked with NASH and significant fibrosis [1,15]. Our analyses confirmed these findings in a logistic regression. On the other hand, it is noteworthy that only a relatively small proportion of the patients who were referred had type 2 diabetes. Based on our design, we were unable to determine the true prevalence of NASH among patients with T2DM in our catchment area. It is possible that some patients with type 2 diabetes were not referred due to age or comorbidities. It is also possible that the symptoms or signs associated with NAFLD could be attributed to type 2 diabetes (e.g., fatigue). Likewise, elevated liver enzymes could be linked with metabolic dysregulation, and this could mean that patients with type 2 diabetes were less likely to be referred. Finally, antidiabetic medication such as semaglutide or liraglutide has been shown to have a possible beneficial effect on patients with NASH [16,17]. It would be very interesting to evaluate this question systematically. In particular, a study assessing the proportion of patients with NASH among patients with type 2 diabetes, followed in the primary sector, could provide essential information. In our study, 31.8% of the 110 patients who underwent a liver biopsy had normal liver enzymes at referral. This underlines that NAFLD screening should not focus solely on routine blood tests but also on diagnostic imaging as well as metabolic risk factors. A previous study found that the implementation of the FIB-4 score prevented 70% of the total NAFLD cases referred to the hepatological department [14].

We found a low proportion (about 40%) of patients with type 2 diabetes. Based on our design, we are unable to determine the true prevalence of NASH among patients with T2DM in our catchment area. It may be that our findings reflect that patients with T2DM have comorbidities, which means that their treating endocrinologist or general practitioner chose not to refer the patient to us. It is also possible that patients with T2DM are receiving medication that has a beneficial effect on their NASH. Antidiabetic medication such as semaglutide or liraglutide has been shown to have a possible beneficial effect on patients with NASH. It would be very interesting to evaluate this question systematically. In particular, a study assessing the proportion of patients with NASH among patients with type 2 diabetes, followed in the primary sector, could provide essential information. Such a study could include different scores, e.g., the BARD score, which was developed based on a retrospective analysis of patients with NAFLD from 2001 to 2005 [18]. Differences between groups of patients with simple steatosis with NASH with or without fibrosis were performed. In addition, NASH with no fibrosis, stage 1–2 fibrosis, or stage 3–4 fibrosis were compared. The BARD score was developed based on characteristics predicting at least a 2.4-fold higher risk of fibrosis. The score includes the variables BMI, AST/ALT Ratio, and Diabetes. The AUCROC for the score was 0.81, and the positive and negative predictive values (NPV) were 43% and 96%, respectively [18]. We found that T2DM was a significant predictor of NASH with significant fibrosis. On the other hand, the fact that we found a lower proportion of patients with T2DM than expected is likely to affect the positive and negative predictive value of the BARD score in our sample.

A small number of referral letters included information about obesity, although this is a risk factor for NASH [1]. Previous evidence shows that obesity is closely linked with NAFLD [19]. It is possible that the link reflects a generalized proinflammatory state [20,21,22]. Visceral adipose tissue is especially important. The pathogenesis is thought to be a multiple-hit process involving insulin resistance, oxidative stress, apoptosis, and adipokines [23]. Obesity is a major risk factor for insulin resistance and type 2 diabetes mellitus and is linked with several disorders including NAFLD as well as obstructive sleep apnea and polycystic ovary syndrome [24]. Insulin resistance has been associated with adipose tissue dysfunction, which can lead to the release of cytokines and adipokines. The disease course depends on several factors including both lifestyle as well as genetic factors [25,26]. Hepatic steatosis can develop as a result of the accumulation of triglycerides in the liver [24,27]. The development of hepatic steatosis occurs at the same time as increased free fatty acids, free cholesterol, and other lipid metabolites, leading to an increased lipotoxicity. This can lead to mitochondrial dysfunction with oxidative stress and hormonal disturbances [28]. The pathogenesis is complex and not yet fully understood.

In our cohort, a large proportion of patients suffered from nausea. In agreement with our findings, a study including data from NHANES found that NAFLD is associated with impairment of the Health-related Quality of Life (HRQOL) [29]. The study found that 22% of patients with NAFLD described their health as ’’poor’’ or ’’fair’’ when compared with 10% of healthy controls. In agreement with these findings, a subsequent study found that patients with NAFLD may experience nonspecific gastrointestinal symptoms with a detrimental impact on their quality of life [30]. The study highlights bloating and abdominal pain, but not nausea. Our study includes a high proportion of patients with significant fibrosis, and this is likely to influence the symptoms at presentation as well as the patients’ overall quality of life [31].

The advantage of the present study is associated with the completeness of the data and follow-up. A large number of patients (more than 1700) were potentially eligible, and only a small number of patients (*n* = 10) with NASH and significant fibrosis declined a liver biopsy. Due to the nature of the electronic patient files, it is unlikely that we have missed patients who ended up with the NAFLD diagnosis. The possible limitation of our study is associated with the external validity. As the Gastro Unit is a specialized department, general practitioners and other departments may be more likely to refer patients for assessment. In fact, several patients were referred with known NASH. It is possible that referral patterns (as well as the diagnostic strategy) will be different when evaluating other departments. We found that NAFLD cases accounted for 18.6% of all referred cases to our department. The findings are likely to reflect the increased awareness but also the increasing prevalence of NAFLD. The number of NAFLD referrals may become an increasing economic burden for the healthcare system [10,32,33]. Considering the rapid increase of obesity and T2DM, the economic burden is expected to continue to increase. Thus, improved diagnostic practices and treatments are urgently needed.

A widely used mechanism for controlling costs in the healthcare system requires a prior assessment and authorization from a general practitioner before patients are referred to a specialist. On the one hand, this gatekeeper function could restrict access to necessary services. On the other hand, the initial assessment made by general practitioners can also help to avoid the overdiagnosis of patients with NAFLD. It is very difficult to assess if the frequency of and nature of specialty referrals is appropriate. There are no explicit criteria to determine if a referral for suspected NAFLD is medically indicated. The exclusion of relevant differential diagnoses is equally important to the exclusion of clinically significant fibrosis. In the absence of criteria, over-utilization and under-utilization could be suspected but not verified, and the optimal referral rate remains unknown. Possible factors that could influence referral include specialist availability, pressure from patients, and diagnostic certainty. Improvement in the diagnostic accuracy of liquid biomarkers and the availability of diagnostic imaging would likely affect the referral of patients.

## Figures and Tables

**Table 1:** Causes of referrals (number and %) and comorbidities described in referral letters of patients with non-alcoholic fatty liver disease (NAFLD). The table shows values for all patients and for patients referred from the primary and secondary sectors.

	All Patients with NAFLD *n* = 323	General Practitioners *n* = 202	Other Hospital Departments *n* = 121
Steatosis on diagnostic imaging	187 (58%)	111 (55%)	76 (63%)
Elevated liver enzymes	220 (68%)	154 (76%)	66 (55%)
Abnormal ferritin levels	27 (8%)	23 (11%)	4 (3%)
Fatigue	32 (10%)	29 (14%)	3 (2%)
Abdominal pain and discomfort	72 (22%)	54 (27%)	18 (15%)

**Table 2:** Differential diagnoses excluded before referral to the Gastro Unit. The table shows the total number and % for all patients and patients referred from the primary and secondary sectors.

	All Patients *n* = 323	General Practitioners *n* = 202	Other Hospital Departments *n* = 121
Alcohol	183 (57%)	108 (53%)	75 (62%)
Drug-induced liver injury	70 (22%)	46 (23%)	24 (20%)
Chronic hepatitis B and C	150 (46%)	90 (45%)	60 (50%)
Haemochromatosis	120 (37%)	72 (36%)	48 (40%)
Autoimmune liver diseases	86 (27%)	44 (22%)	42 (35%)
Alpha-1 antitrypsin deficiency	19 (6%)	8 (22%)	11 (9%)
Wilsons disease	14 (4%)	4 (2%)	10 (8%)
Coeliac disease	10 (3%)	10 (5%)	0 (0%)

**Table 3:** Univariable logistic regression analyses (adjusted for age and gender) of possible predictors of NASH with significant fibrosis in 110 patients who underwent a liver biopsy. The table shows the odds ratios (OR) with 95% confidence intervals (CIs) and *p*-values.

Variable	OR (CIs) Adjusted Gender and Age	*p*-Value	OR (CIs) Multivariable-Adjusted ^1^	*p*-Value
Comorbidities				
Obesity, *n* = 74	1.24 (0.75–2.05)	0.408	1.11 (0.66–1.90)	0.682
Type 2 diabetes, *n* = 46	2.65 (1.53–4.78)	0.00033	2.70 (1.53–4.78)	0.000603
Hypertension, *n* = 53	1.70 (0.99–2.92)	0.054	1.35 (0.75–2.41)	0.312
Dyslipidaemia, *n*= 69	1.00 (0.62–1.65)	0.972	0.81 (0.48–1.38)	0.444
Symptoms				
Abdominal pain and discomfort, *n* = 27	0.77 (0.45–1.31)	0.344	0.64 (0.35–1.14)	0.141
Fatigue, *n* = 13	0.74 (0.35–1.48)	0.409	0.93 (0.43–1.93)	0.845
Nausea, *n* = 9	2.34 (0.85–6.61)	0.097	3.14 (1.05–9.63)	0.040

^1^ The multivariable-adjusted model included all factors in the same logistic regression.

## Data Availability

Data available on request from the corresponding author.
